# The Pharmaceutical Industry in 2022: An Analysis of FDA Drug Approvals from the Perspective of Molecules

**DOI:** 10.3390/molecules28031038

**Published:** 2023-01-20

**Authors:** Beatriz G. de la Torre, Fernando Albericio

**Affiliations:** 1 Kwazulu-Natal Research Innovation and Sequencing Platform, College of Health Sciences, University of KwaZulu-Natal, Durban 4001, South Africa; 2 School of Chemistry and Physics, University of KwaZulu-Natal, Durban 4001, South Africa; 3 CIBER-BBN, Networking Centre on Bioengineering, Biomaterials and Nanomedicine, Department of Organic Chemistry, University of Barcelona, 08028 Barcelona, Spain

**Keywords:** antibodies, biologics, chemical entities, fluorine-based drugs, imaging, natural products, new chemical entities, oligonucleotides, peptides, TIDES

## Abstract

While 2021 ended with the world engulfed in the COVID-19 Omicron wave, 2022 has ended in almost all countries, except China, with COVID-19 being likened to the flu. In this context, the U.S. Food and Drug Administration (FDA) has authorized only 37 new drugs this year compared to an average of 52 in the last four years. Thus 2022 is the second lowest harvest after 2016 in the last six years. This ranking may be transient and will be confirmed in the coming years. In this regard, the reduction in the number of drugs accepted by the FDA this year applies only to the so-called small molecules as there has been no variation in the respective numbers of biologics or TIDES (peptides and oligonucleotides). Monoclonal antibodies (mAbs) continue to be the class with the most drugs authorized (9), while proteins/enzymes (5) and an antibody–drug conjugate complete the biologics harvest. In 2022, five TIDES and seven drugs inspired by natural products have received the green light, thus showing the same tendency as in previous years. Finally, pharmaceutical agents with nitrogen aromatic heterocycles and/or fluorine atoms continue to be predominant among small molecules this year. Furthermore, three drugs have been approved for imaging, reinforcing the trend in recent years for this class of treatments. A keyword in 2022 is bispecificity since four drugs have this property (two mAbs, one protein, and one peptide). Herein, the 37 new drugs approved by the FDA in 2022 are analyzed. On the basis of chemical structure alone, these drugs are classified as the following: biologics (antibodies, antibody-drug conjugates, proteins/enzymes), TIDES (peptide and oligonucleotides), combined drugs, natural products; nitrogen aromatic heterocycles, fluorine-containing molecules, and other small molecules.

## 1. Analysis

A year ago, a question mark was hanging over the world, and under this uncertainty, the future of the pharmaceutical industry was in doubt. We were submerged in a wave of the COVID-19 pandemic produced by the Omicron variant, and we had no clear understanding of how the approved vaccines would respond to the new variant and, therefore, of how “normality” could be achieved again. Right now, a year later, although the situation in China is not entirely clear, the vast majority of countries have been able to efficiently manage the COVID-19 pandemic, and this disease is increasingly treated in a similar way to others such as influenza (flu), which also causes a large number of deaths worldwide every year. Although many pharmaceutical companies have launched vaccine programs since the start of the COVID-19 pandemic back in 2020, only two vaccines, both mRNA-based, produced by Pfizer-BioNTech and Moderna, are used in most countries, except for China, Russia, and their areas of influence. This achievement has boosted the standing of mRNA technology for the development of vaccines for other diseases, including various types of cancer. 

As expected, the number of drugs accepted by the FDA in 2020 and 2021 with respect to previous years was not affected by the pandemic: 50 in 2021, 53 in 2020, 48 in 2019, and 59 in 2018 [1,2]. The maintenance of the numbers is attributed to the slow implementation processes of the pharmaceutical industry, which make any major change take years to be reflected in the number of drugs accepted. However, a question is raised regarding the significant decrease in the number of drugs approved in 2022 (only 37) [3]: are we entering a valley in the drug discovery arena, or is this figure temporary? This question will be answered in the coming years.

**Figure 1:**
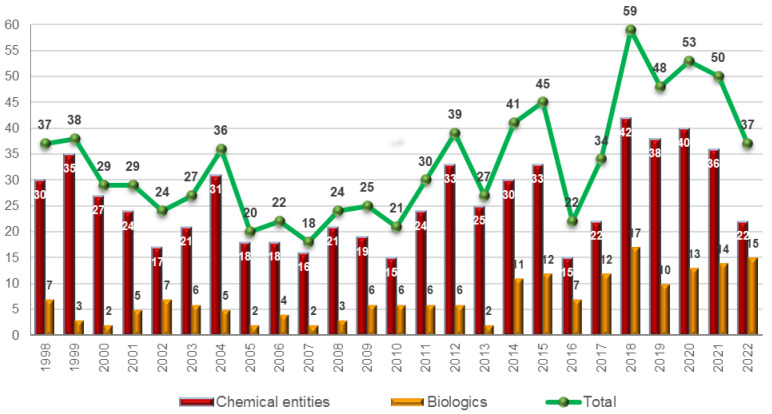
Drugs (new chemical entities and biologics) approved by the FDA in the last 25 years. Adapted with permission from Ref. [1]. Copyright 2022, copyright MDPI [1,2,3].

The 37 drugs approved this year are divided between 15 biologics (14, 13, 10, and 17 in 2021, 2020, 2019, and 2018, respectively) [2,3] and 22 new chemical entities (NCEs), including hyperpolarized Xe-129 in this last category (36, 40, 38, and 42 in 2021, 2020, 2019, and 2018, respectively) (Figure 1) [2,3]. While the number of biologics approved is in full agreement with the tendency shown by this category over recent years, there are significantly fewer NCEs (22 vs. 37 on average, which represents only 55%). This year, biologics account for 40% of all drugs, when in recent years this figure was approximately 27% (96 biologics out of a total of 352 drugs since 2014) [2]. 

This year, the Center for Biologics Evaluation and Research (CBER) has added 12 new Biological License Application Approvals, a similar figure to 2021, when 13 were registered [4]. While in 2021, COMIRNATY^TM^, the Pfizer-BioNTech COVID-19 vaccine, was fully approved, this year it was the turn of SPIKEVAX^TM^, the COVID-19 vaccine developed by Moderna [4]. To date, these are the only two COVID-19 vaccines that have been fully authorized by the FDA. In this regard, in 2021, the Novavax and Janssen COVID-19 vaccines received only the status of Emergency Use Authorization (EUA) [5].

## 2. Discussion

Table 1 shows the fifteen biologics that have been approved in 2022, of which nine are monoclonal antibodies (mAbs), one an antibody–drug conjugate (ADC), and five proteins, namely a toxin, a fusion protein, a hormone (glycoprotein), and two enzymes (Table 1).

Regarding biologics, one of the highlights of this year has been the approval of three drugs, namely two mAbs and one fusion protein, that show bispecificity. Thus, these drugs can simultaneously bind to two distinct epitopes on one antigen or to two different antigens. Teclistamab-cqyv (Tecvayli^TM^), which targets the CD3 receptor expressed on the surface of T cells and B cell maturation antigen (BCMA) expressed on the surface of malignant multiple myeloma B-lineage cells, is used for the treatment of relapsed/refractory multiple myeloma. Faricimab-svoa (Vabysmo^TM^) is indicated for the treatment of neovascular and diabetic macular degeneration, and it targets vascular endothelial growth factor and angiopoietin 2. The fusion protein tebentafusp-tebn (Kimmtrak^TM^), which is a bispecific gp100 peptide-HLA-directed CD3 T cell engager, is indicated for the treatment of unresectable/metastatic eye cancer. In previous years, three other bispecific antibodies were approved by the FDA [2]. In 2014, blinatumomab (Blincyto^TM^), targeting CD19 and CD3, was authorized for the treatment of Philadelphia chromosome-negative B cell acute lymphoblastic leukemia. Four years later, in 2018, emicizumab (Hemlibra^TM^), which targets clotting factors IXa and X, was approved for hemophilia A. Finally, in 2021, amivantamab-vmjw (Rybrevant^TM^), which targets epidermal growth factor and MET receptors, received authorization for the treatment of cancer. 

In addition to the two aforementioned bispecific mAbs approved this year, seven more mAbs received the green light, together accounting for 24% (9 vs. 37) of all the drugs accepted by the FDA in 2022. 

Interestingly, Opdualag^TM^ is a combination of two mAbs (nivolumab and relatlimab-rmbw), and it is used for the treatment of unresectable/metastatic melanoma. Nivolumab (Opdivo^TM^) is also commercialized alone for a large number of cancer-related diseases. 

Cancer continues to be the first target for mAbs. In addition to the two mAbs previously discussed, three more have been approved for cancer this year, namely mirvetuximab soravtansine-gynx (Elahere^TM^), tremelimumab-actl (Imjudo^TM^), and osunetuzumab-axgb (Lunsumio^TM^). However, following the trend of previous years, mAbs have been approved for others targets. In addition to faricimab-svoa (Vabysmo^TM^) for macular degeneration, authorization has been given to spesolimab-sbzo (Spevigo^TM^) for psoriasis, teplizumab-mzwv (Tzield^TM^) for diabetes type 1, and ublituximab-xiiy (Briumvi^TM^) for relapsed multiple sclerosis.

On a negative note regarding mAb, 2022 has seen controversy around aducanumab-avwa (Aduhelm^TM^), which was approved last year for the treatment of Alzheimer’s disease [1,2]. Although the clinical phase IV for this drug is still underway, the market prospects are in line with concerns about its effectiveness. However, as often happens in the roller coaster ride of drug discovery, Biogen, the company that developed aducanumab-avwa (Aduhelm^TM^), associated with Eisai, announced in November this year that another mAb, lecanemab, showed success in clinical trials and their intention of securing its approval by the FDA in 2023 [6].

**Figure 2:**
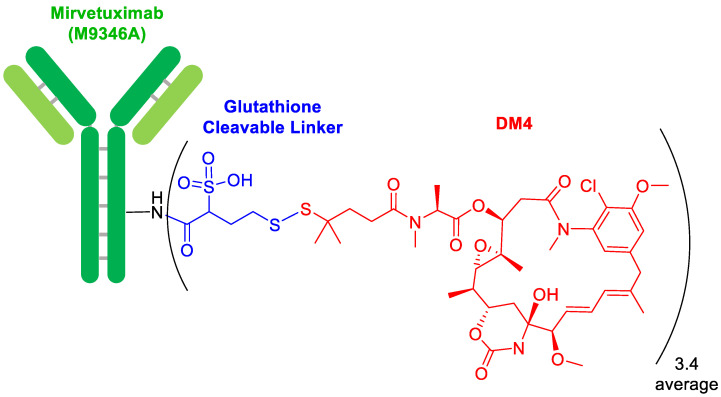
Structure of mirvetuximab soravtansine-gynx.

In terms of ADCs, 2022 has continued with a trickle of this type of drug. Thus, mirvetuximab soravtansine-gynx (Elahere^TM^) (Figure 2) for ovarian cancer has been approved, being the 14th of this class on the market. This ADC contains as a payload drug mersantine, an analog of maytansine (DM4), which is a microtubule-targeted cytotoxic agent also present in rastuzumab emtansine (Kadcyla^TM^), which was approved by the FDA in 2013 [2]. Mersantine is linked to the Lys of the anti-folate receptor 1 through a cleavable linker formed by a disulfide bridge, whose lability to glutathione is modulated by two methyl groups in the α-position of the disulfide bridge. In addition, the linker contains a sulfonic acid moiety, which confers hydrophilicity to the linker to facilitate bioconjugation. 

In addition to the fusion protein tebentafusp-tebn (Kimmtrak^TM^), four more proteins have been approved this year. Eflapegrastim-xnst (Rolvedon^TM^) is a hormone (glycoprotein) that prevents infections in adults with febrile neutropenia associated with myelosuppressive anti-cancer drugs for the treatment of non-myeloid malignancies. It is a long-acting granulocyte colony-stimulating factor (G-CSF) analog that produces granulocytes and stem cells to be released into the bloodstream by stimulation of the bone marrow. Olipudase alfa-rpcp (Xenpozyme^TM^) is an enzyme to treat acid sphingomyelinase deficiency (ASMD, Niemann-Pick disease), a metabolic genetic disorder that causes brain damage and swelling of organs such as the spleen and liver. Treatment with this drug can improve the function of affected organs and tissues, based on clinical trials. At the end of the year, anacaulase-bcdb (NexoBrid^TM^), which is a mixture of proteolytic enzymes isolated from the pineapple plant, was approved for the debridement (removal of eschar, which is dead and damaged tissue) of severe burn wounds.

This year another botulinum toxin, daxibotulinumtoxinA-lanm (Daxxify^TM^), has been authorized for the temporary improvement of the appearance of eyebrows. This molecule joins other toxins from the same family that were authorized by the FDA in previous years [2], such as prabotulinumtoxin (Jeuveau^TM^), approved in 2019 for a similar indication; abobotulinumtoxinA (Dysport^TM^), approved in 2016 for the treatment of lower-limb spasticity in pediatric patients; and onabotulinumtoxinA (Botox^TM^), approved in 2010 for chronic migraine in prophylactic treatments. When referring to a lethal toxin, it is important to highlight that misadministration can lead to the paralysis of unintended muscles, with resulting cosmetic and health problems. 

TIDES (oligonucleotides and peptides) are chemical entities and are therefore associated with the simple chemical concept of “pure substance”, which implies a deep characterization, including minor impurities. However, in most cases, they are macromolecules similar to biologics. This year, five TIDES (four peptides and one oligonucleotide) have been approved, which account for almost 15% (5 vs. 37) of the drugs approved. In this report, those drugs which contains peptidomimetics are also considered peptides.

**Figure 3:**
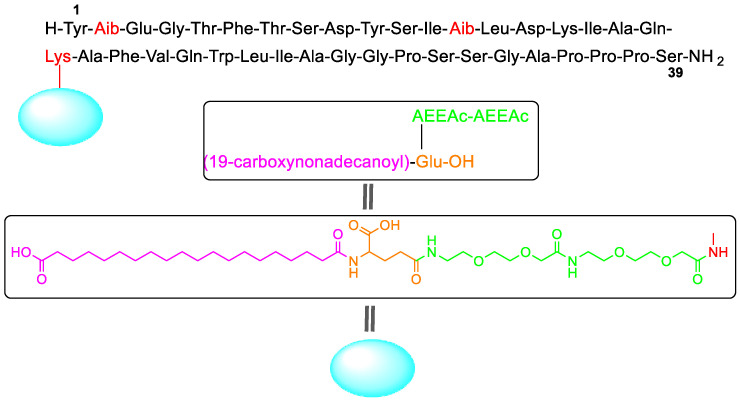
Structure of tirzepatide.

Tirzepatide (Mounjaro^TM^) (Figure 3) has been authorized for the treatment of type 2 diabetes. It is also showing excellent results for the management of obesity and non-alcoholic steatohepatitis (NASH), thus making it a potential blockbuster drug. It is a GIP (gastric inhibitory polypeptide) analog that activates GIP and GLP-1 (glucagon-like peptide-1) receptors, thus being a first-in-class drug. Comprising 39 amino acids, tirzepatide is one of the largest synthetic peptides on the market. It has a lipidated sidechain pending at a Lys residue situated in the middle of the backbone (position 20). The sidechain is formed by eicosanedioic acid (C20 diacid) linked through the γ-carboxylic group of the Glu residue and two mini-polyethyleneglycol (PEG) moieties [(2-(2-aminoethoxy)ethoxy)acetic acid] to the ε-amino of the Lys. Two amino acids of the backbone are aminoisobutyric acid (Aib). These two residues and the sidechain confer tirzepatide stability to metabolism. Similar moieties are present in other long-term stable peptides such as semaglutide and liraglutide, which are also GLP-1 agonists [2]. 

**Figure 4:**
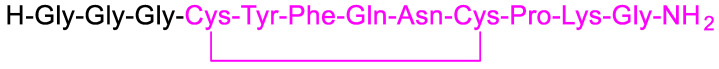
Structure of terlipressin (in pink the sequence of lypressin).

Terlipressin (Terlivaz^TM^) (Figure 4), which is a vasopressin receptor agonist (analog of vasopressins), has been authorized as a treatment to improve kidney function in adults with hepatorenal syndrome affected by a rapid decrease in kidney function. It is also indicated for the management of low blood pressure. Structurally, terlipressin contains an extra three Gly residues to the N-terminal of lysine vasopressin (lypressin). 

**Figure 5:**
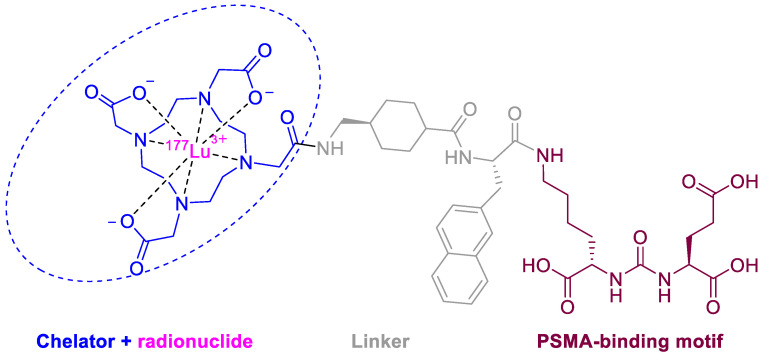
Structure of 177Lu vipivotide tetraxetan.

The last few years have witnessed a considerable increase in the number of radiopharmaceuticals launched onto the market [2]. One such drug has been approved by the FDA this year. Lutetium (177Lu) vipivotide tetraxetan (Pluvicto^TM^) (Figure 5) is indicated for the treatment and detection of prostate-specific membrane antigen (PSMA)-positive metastatic castration-resistant prostate cancer. This is the third PSMA-targeted positron emission tomography (PET) imaging drug approved after piflufolastat F-18 (Pylarify^TM^) last year and Ga-68 PSMA-1 two years ago [2]. These three drugs share the same PSMA-specific pharmacophore, the urea of the two α-amino functions of L-Glu and L-Lys. In the case of 177Lu vipivotide tetraxetan, the ε-amino function of the Lys residue is anchored to the Lu-DOTA (1,4,7,10-tetraazacyclododecane-1,4,7,10-tetraacetic acid) complex through the 2-naphthyl-L-Ala-trans-cyclohexyl linker. It is the second 177Lu-based theranostic after Lu 177 DOTA-TATE (Lutathera^TM^), which was approved in 2018.

**Figure 6:**
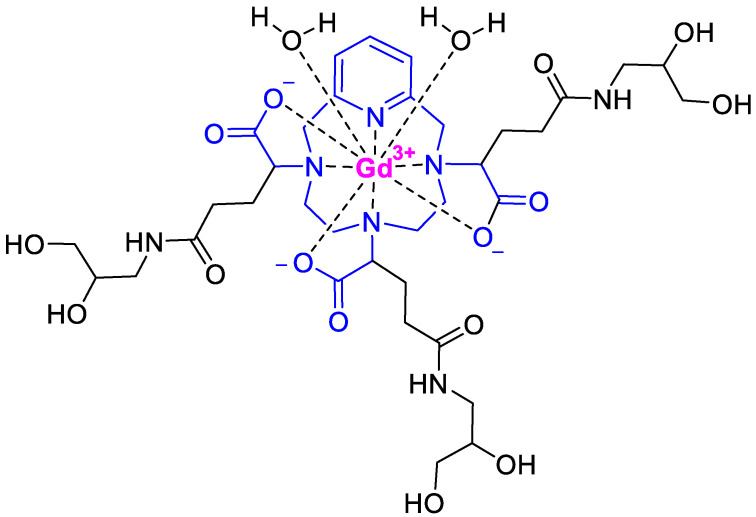
Structure of gadopiclenol.

Gadopiclenol (Elucirem^TM^) (Figure 6) is a magnetic resonance imaging (MRI) contrast agent used to detect abnormal vascularity in the central nervous system (CNS) and also in the body. It is a Gd chelate of a pyclen-based macrocyclic structure {2,2′,2′′-(3,6,9-triaza-1(2,6)-pyridinacyclodecaphane-3,6,9-triyl)tris(5-((2,3-dihydroxypropyl)amino)-5-oxopentanoic acid)]. Gadopiclenol (Elucirem^TM^) exhibits high relaxivity, no protein binding, high kinetic inertness, and a good pharmacokinetic profile. 

In this section of imaging treatments, Xenoview, which is hyperpolarized Xe-129, an MRI agent, has been approved for the evaluation of pulmonary function and imaging. 

**Figure 7:**
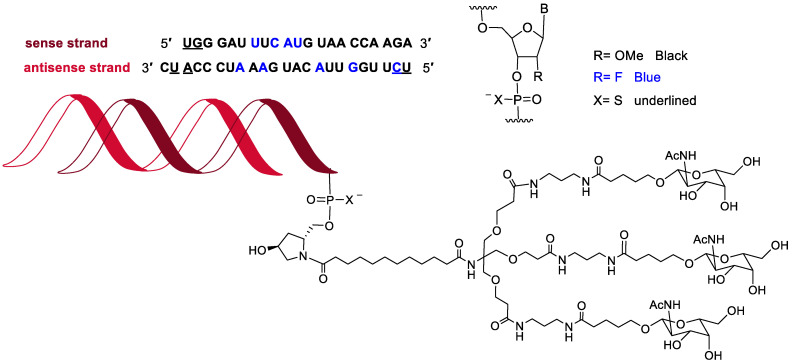
Structure of vutrisiran.

In the last seven years (2016–2022) [2], 12 oligonucleotide-based drugs have been granted authorization by the FDA, which account for 80% of the total of this class on the market (15 oligo drugs on the market regulated by the FDA). This year, vutrisiran (Amvuttra^TM^) (Figure 7) received the green light for the treatment of the polyneuropathy of hereditary transthyretin-mediated amyloidosis in adults. The chemical structure of vutrisiran is very similar to the following drugs: inclisiran, which was approved last year for atherosclerotic cardiovascular disease and familial hypercholesterolemia; lumasiran, which was approved in 2020 for hyperoxaluria type 1; and givosiran, approved in 2019 for acute hepatic porphyria [2]. Thus vutrisiran is a double-stranded siRNA, with 21 and 23 ribonucleosides for the sense and antisense strands, respectively. It has a total of six tiophosphate linkages—the same as those oligonucleotide-based drugs previously approved—as well as nine 2′-F-ribonucleoside units (12, 10, and 16 in inclisiran, lumasiran, and givosiran, respectively) to improve the stability of the double-strand. The remaining ribonucleosides are 2′-methoxy. Like those previously approved, vutrisiran is presented through an Enhanced Stabilization Chemistry (ESC), which, in addition to F- and methoxy-ribonucleosides, has the 3′ end of the sense strand linked to a trivalent N-acetylgalactosamine (GalNAc), which mediates the binding and internalization of the drug by hepatocytes. The six thiophosphates are in the other three ends—two in each. This kind of structure is also present in inclisiran, lumasiran, and givosiran [2]. 

In 2022, three drugs that contain more than one active pharmaceutical ingredient (API) have been approved. Thus, in addition to Opdualag^TM^, which contains two mAbs (Nivolumab and Relatlimab-rmbw), Voquezna™ Triple Pak™ (vonoprazan, amoxicillin, clarithromycin) and Relyvrio™ (sodium phenylbutyrate, taurursodiol) have been launched onto the market. 

**Figure 8:**
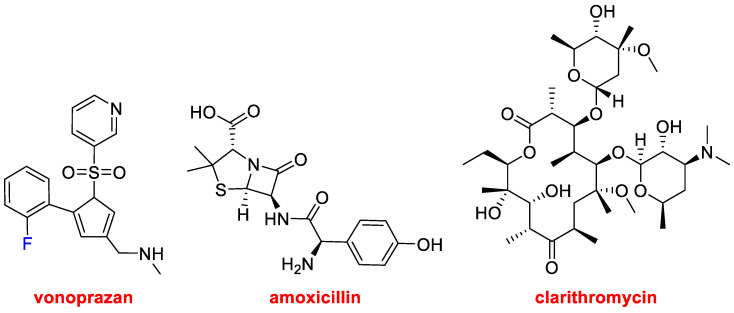
Structure of the components of the combination drug Voquezna™ Triple Pak™.

Voquezna™ Triple Pak™ (Figure 8) and its simpler version Voquezna™ Dual Pak™, with only vonoprazan and amoxicillin, are indicated for the treatment of *Helicobacter pylori* infection in adults. In addition to the antibiotic(s), vonoprazan in the form of fumarate, which is a potassium-competitive acid blocker (PCAB), is added for its potent acid-inhibiting effects, as *Helicobacter pylori* colonizes and flourishes in a low pH environment. Amoxicillin (approved in 1974) inhibits bacterial wall synthesis, but it does not eliminate *Helicobacter pylori* by itself. On the other hand, clarithromycin (approved in 1990) is not used alone because, although active against this bacterium, it promotes resistance. 

**Figure 9:**
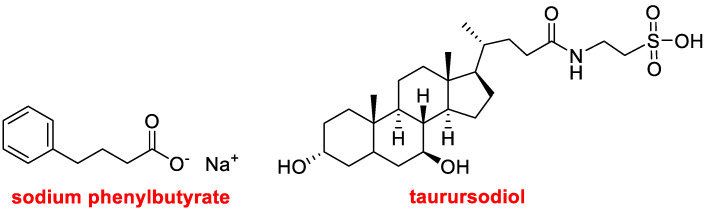
Structure of the components of the combination drug Relyvrio^TM^.

Relyvrio^TM^ (Figure 9), which contains sodium phenylbutyrate and taurursodiol, is a combination drug indicated for the treatment of amyotrophic lateral sclerosis (ALS). Sodium phenylbutyrate is a chemical chaperone that prevents protein aggregation—a process that may lead to cell death. It was approved by itself by the FDA in 1996 for the treatment of genetic diseases. Taurursodiol, also known as ursodoxicoltaurine or tauroursodeoxycholic acid, is a hydrophilic bile acid that reduces apoptotic effects.

With these three combination drugs, 19 such drugs have been authorized by the FDA between 2016 and 2022 [2]. This figure highlights the importance of this drug discovery strategy, which takes advantage of “old” drugs, in many cases already on the market. 

**Figure 10:**
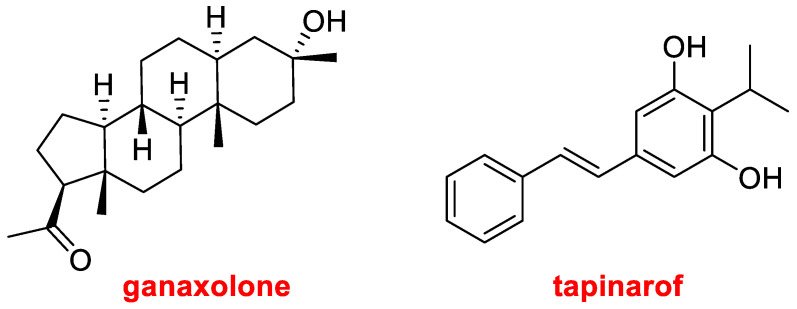
Structures of ganaxolone and tapinarof, both drugs inspired by natural products.

Natural products have always been an inspiration for drug discovery. Thus, in addition to the biologics, it is important to highlight the mixture of proteolytic enzymes from the pineapple plant, anacaulase-bcdb for the treatment of burns, TIDES (the oligo and the two canonical peptides), amoxicillin derived from penicillin G, clarithromycin from erythromycin, and taurursodiol. This year two more drugs of the natural product class have been approved. Ganaxolone (Ztalmy^TM^) (Figure 10) is another steroid to treat seizures in people with cyclin-dependent kinase-like 5 deficiency disorder. Tapinarof, also named Benvitimod (Vtama^TM^) (Figure 10), is indicated for plaque psoriasis and has been found in bacterial symbionts of nematodes.

**Figure 11:**
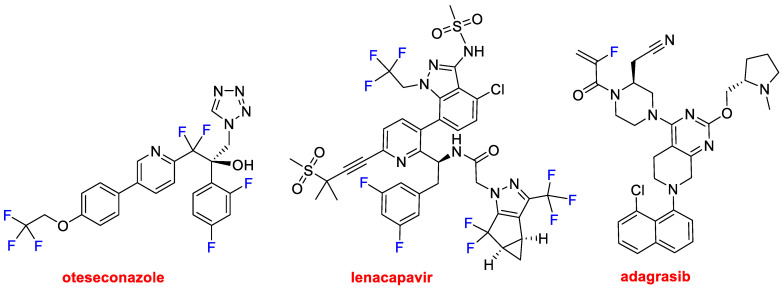
Structures of the F containing drugs: oteseconazole, lenacapavir, and adagrasib.

Fluorine is a key atom in drug discovery as it is present in many pharmacological agents. In recent years, approximately half of all small molecules contain at least one F and very often a CF_3_ moiety. This year, in addition to the oligonucleotide-based drug vutrisiran, four more APIs containing fluorine have been approved. Thus, as well as vonoprazan (one of the constituents of Voquezna™), three more drugs belonging to this class have been released onto the market. Oteseconazole (Vivjoa™) and lenacapavir (Sunlenca™) (Figure 11) could be considered F-rich molecules because they contain trifluoro and difluoro methyl moieties and F atoms bound to aromatic rings. Oteseconazole is indicated for the recurrence of the infection caused by vulvovaginal candidiasis. Lenacapavir is an antiretroviral for treating HIV in individuals in which other treatments have failed. It is a first-in-class drug that blocks the capsid of HIV-1, thus interfering with other steps of the viral lifecycle. It is also being investigated as a prophylactic. Adagrasib (Krazati™) (Figure 11) is indicated for the treatment of metastatic or KRAS G12C-mutated locally advanced non-small cell lung cancer in adults who have received at least one prior systemic therapy.

Among small molecules, there is no doubt that the most common drugs on the market are those that contain nitrogen aromatic heterocycles. This year, in addition to the four drugs that contain F atoms, nine more drugs belonging to this class have been approved. Therefore, approximately two-thirds of the small molecules (13 vs. 19) contain this chemical moiety. Various of these drugs are kinase-related. 

**Figure 12:**
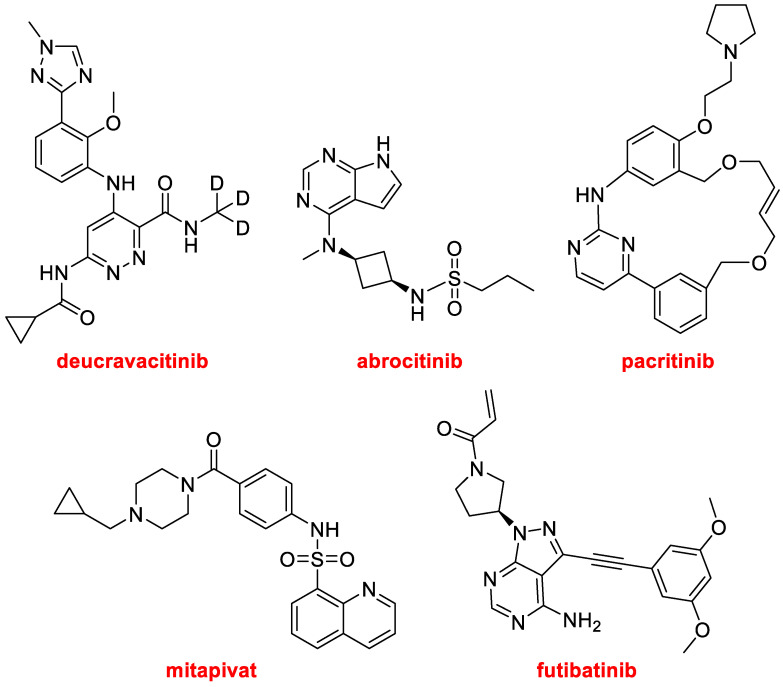
Structures of the kinase-related drugs deucravacitinib, abrocitinib, pacritinib, futibatinib, and mitapivat.

Deucravacitinib (Sotyktu™) (Figure 12), for the treatment of psoriasis, is a selective allosteric inhibitor of non-receptor tyrosine-protein kinase. It contains a deuterated methyl amide moiety, and it is the second deuterated drug approved by the FDA after deutetrabenzine (Austedo™), which was authorized in 2017 for the treatment of chorea associated with Huntington’s disease [2]. 

Abrocitinib (Cibinqo™) (Figure 12), which is a sulfonamide, is a Janus kinase inhibitor for the treatment of eczema (atopic dermatitis). The macrocycle pacritinib (Vonjo™), (Figure 12) also a Janus kinase inhibitor, is indicated for the treatment of myelofibrosis, an uncommon bone marrow cancer. Futibatinib (Lytgobi™) (Figure 12), a tyrosine kinase inhibitor, has been approved for the treatment of bile duct cancer (cholangiocarcinoma). Finally, in this section of kinase-related drugs, mitapivat (Pyrukynd™) (Figure 12), which is also a sulfonamide, is a pyruvate kinase activator, used to treat hemolytic anemia. 

**Figure 13:**
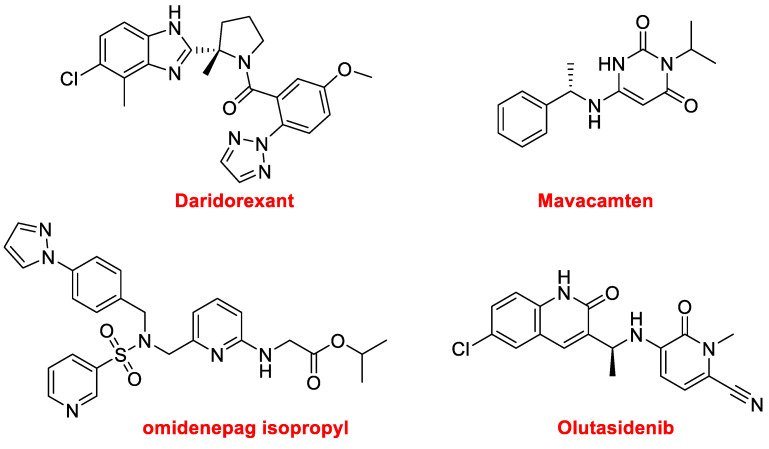
Structures of additional nitrogen aromatic heterocycle-based drugs.

Four additional nitrogen aromatic heterocycles have been approved this year. Daridorexant, formerly known as nemorexant (Quviviq™) (Figure 13), for the treatment of insomnia, is an orexin antagonist. Mavacamten (Camzyos™) (Figure 13), which is a cardiac myosin inhibitor, is used to treat obstructive hypertrophic cardiomyopathy. The sulfonamide, omidenepag isopropyl (Omlonti™), an ophthalmic solution (Figure 13), is used to reduce elevated intraocular pressure in patients with ocular hypertension or open-angle glaucoma. Finally, olutasidenib (Rezlidhia™) (Figure 13) is an oncologic drug used to treat relapsed or refractory acute myeloid leukemia.

## 3. Conclusions and Perspectives

*Health* and *well-being* have been magic words for society throughout history. In this context, the COVID-19 pandemic has served to highlight the importance of having a well-structured and solid health ecosystem available to the entire population. In this regard, the role played by pharmaceutical companies is fundamental. For the middle classes, these companies have ceased to be distant entities and are recognized as being part of the solution, with positive connotations in many aspects but also some negative ones. The pharmaceutical sector has acted with rigor and efficiency during the pandemic and has entered millions of homes, with brand names and even the names of some of the key researchers becoming known, thus bringing about even greater responsibility for the sector.

Regarding the treatment of COVID-19, it can be concluded that this year there have been no major developments. The only two fully FDA-approved vaccines, Pfizer-BioNTech and Moderna, are prevalent, and the FDA has not granted EUA to new treatments. Thus, only the antivirals molnupiravir (Lagevrio^TM^), remdesivir (Veklury^TM^) (nucleoside and nucleotide analogues, respectively), and Paxlovid^TM^, which contains nirmatrelvir and the ritonavir, have received authorization for the treatment of COVID-19 in individuals at high risk of progressing to severe status [7]. Furthermore, the long-acting antibody combination Evusheld^TM^ (co-package of tixagevimab with cilgavimab) has been authorized as a preventative treatment [7]. For immunocompromised people with COVID-19, the Center for Disease Control and Prevention (CDC) recommends convalescent plasma [8], which uses blood from individuals who have recovered from COVID-19 to treat others. The webpage of the CDC even recommends over-the-counter drugs such as acetaminophen or ibuprofen to alleviate the symptoms of COVID-19.

In the context of the continuing COVID-19 pandemic, if we were to assess the strength of the pharmaceutical industry in 2022 based on the number of new drugs accepted by the FDA, the conclusion could be worrying as the number has been significantly lower than in previous years (37 vs. an average of 52.5 for the last 4 years) [2]. However, it should be considered that the process of putting a drug onto the market is slow and that this number may therefore reflect only a slowdown in bureaucratic and administrative processes. In any case, this relative decrease in the number of drugs should be attributed more to small molecules than to biologics, since the 15 biologics accepted represent the second-best harvest after 2018, when 17 were authorized [2].

**Figure 14:**
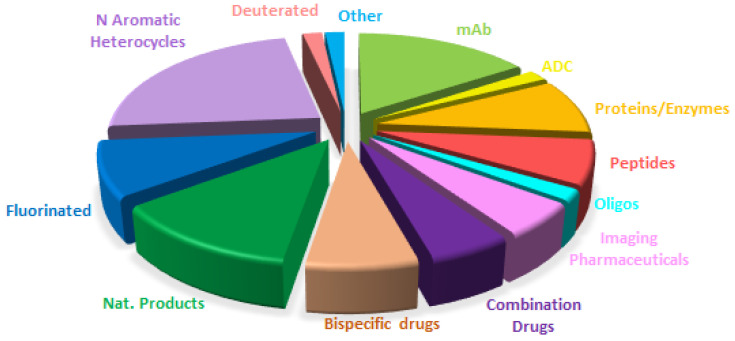
Drugs approved by the FDA in 2021 classified on the basis of chemical structure (drugs can belong to more than a class) Adapted with permission from Ref. [1]. Copyright 2022, copyright MDPI.

Figure 14 shows a breakdown of FDA approvals this year based on the chemical structure of the drugs.

It is important to highlight that four drugs, namely two mAbs (teclistamab-cqyv (Tecvayli^TM^) and faricimab-svoa (Vabysmo^TM^)), one fusion protein (tebentafusp-tebn (Kimmtrak^TM^)), and a peptide (tirzepatide (Mounjaro^TM^)) show bispecifity, targeting two different antigens or receptors. In contrast to 2021, this year has not seen the authorization of any true pegylated drugs. Only the peptide tirzepatide (Mounjaro^TM^), which contains two mini PEGs in its side-chain, has received the green light.

**Figure 15:**
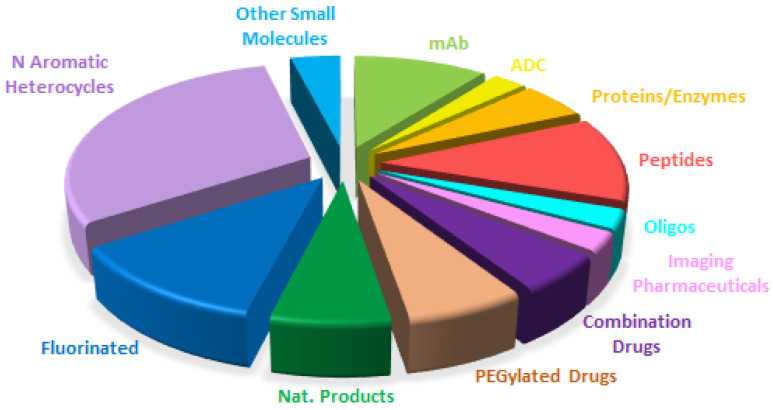
Similar to Figure 14 for the year 2021, taken with permission from reference [1]. Copyright 2022, copyright MDPI.

Overall if Figure 14 is compared with Figure 15, which shows the drugs approved in 2021 (the colors for each class have been kept with the exception that bispecific drugs have substituted PEGylated drugs), the distribution seems very similar, with the growth of biologicals to the detriment of small molecules. This shows the maturity of the sector with well-established trends.

Once again, in 2022, biologics have maintained double-digit status. Of the nine mAbs approved, five are for cancer, but the remaining four are indicated for very diverse targets, from diabetes type 1 to macular degeneration, and relapsed multiple sclerosis to psoriasis. These figures reflect the expansion of this class of drugs for diseases other than cancer, which was the major indication of this class for many years. Next year, lecanemab is expected to be approved for the treatment of Alzheimer’s disease. This will be the second mAb for this target after aducanumab-avwa (Aduhelm^TM^), authorized in 2021, and for which the results are not entirely conclusive.

Another ADC, namely soravtansine-gynx (Elahere^TM^), has been approved this year. A distinctive feature of this drug is the reversible linker, which is a disulfide bridge flanked by a dimethyl moiety to ensure better stability to the glutathione, and sulfonic acid to increase the hydrophilicity of the linker.

Furthermore, in 2022, five proteins have been approved, among them a third botulinum toxin daxibotulinumtoxinA-lanm (Daxxify^TM^). It is interesting to highlight the presence of anacaulase-bcdb (NexoBrid^TM^), a mixture of proteolytic enzymes isolated from the pineapple plant, which can fall in the traditional or popular medicine category.

In addition, four peptides and one oligonucleotide have been given the green light, tirzepatide (Mounjaro^TM^) being the most outstanding peptide for its complexity (39 amino acids with an additional sidechain containing a long fatty acid), but more important for its indications: diabetes type 2 and the control of the obesity. The second peptide approved, terlipressin (Terlivaz^TM^), is a simple disulfide cyclic peptide with three Gly residues added to the well-known and studied lysine vasopressin structure.

The oligonucleotide approved, vutrisiran, is a double-stranded siRNA, very similar to inclisiran, lumasiran, and givosiran, which were authorized in recent years. In addition to the double strand, all these drugs have the same structural features to confer stability, namely tiophosphate linkages, F and methoxy ribonucleoside units, and the ESC, which is a short dendrimer bearing N-acetylgalactosamine. It is expected that more members of this class of drugs will reach the market in the coming years.

In 2022, the use of hyperpolarized Xe-129 (Xenoview^TM^) was authorized for MRI, thus showing that the use of hyperpolarized noble gasses (He-3 and Xe-129) is an emerging opportunity for diagnostics.

In the natural product section, this year has witnessed the approval of seven drugs, namely the botulinum toxin daxibotulinumtoxinA-lanm, anacaulase-bcdb, which is a mixture of proteolytic enzymes from the pineapple plant, and five small molecules, two of them belonging to the steroid class, which is one of the most frequent on the market. 

If biologics and peptides are excluded from the analysis, a large majority of the drugs approved this year are either nitrogen aromatic heterocycle- or fluorine-based. In many cases, both moieties are present in the same drug.

The presence of nitrogen aromatic heterocycle- and fluorine-based drugs continues to be a constant. Thus, if biologics and peptides are excluded from the analysis, 82% of the drugs approved this year contain the first moiety and 33% the second.

Although oncology continues to be the main indication of the drugs approved by the FDA, this year, interestingly, dermatology is the second one.

We consider that our honorific title “Molecule of the Year 2022” should go to the peptide tirzepatide (Mounjaro^TM^), which is a bispecific molecule GIP and GLP-1 receptors, being a first-in-class drug. Several peptides on the market mimic GLP-1, but this is the first one that also mimics GIP. This is also the first fully synthetic peptide launched by Eli Lilly through a hybrid solid-phase synthesis (synthesis of the three protected fragments) and solution synthesis (assembly on the protected fragments and final global deprotection) approach. The sales are forecasted to reach more than $10 billion.

## Figures and Tables

**Table 1:** Biologics approved by the FDA in 2022 [3].

Trade Name ^a^	Active Ingredient ^a^	Class	Indication
Briumvi^TM^	Ublituximab--xiiy	Monoclonal Antibody	Relapsed multiple sclerosis
Daxxify^TM^	DaxibotulinumtoxinA-lanm	Protein (toxin)	Temporary improvement of moderate/severe glabellar lines (eyebrows) associated with corrugator and/or procerus muscle activity
Elahere^TM^	Mirvetuximab soravtansine-gynx	Antibody Drug Conjugate	Fallopian tube/primary peritoneal cancer resistant to platinum therapy
Enjaymo^TM^	Sutimlimab-jome	Monoclonal Antibody	Alleviation of red blood cell transfusion due to autoimmune hemolytic anemia
Imjudo^TM^	Tremelimumab-actl	Monoclonal Antibody	Unresectable hepatocellular carcinoma
Kimmtrak^TM^	Tebentafusp-tebn	Bispecific Fusion Protein	Unresectable/metastatic uveal melanoma (eye cancer)
Lunsumio^TM^	Osunetuzumab-axgb	Monoclonal Antibody	Relapsed/refractory follicular (non-Hodgkin) lymphoma in adults
NexoBrid^TM^	Anacaulase-bcdb	Mixture of proteolytic enzymes	Debridement of burn wounds
Opdualag^TM^	Nivolumab & Relatlimab-rmbw	Combination of Monoclonal Antibodies	Unresectable/metastatic melanoma
Rolvedon^TM^	Eflapegrastim-xnst	Hormone (glycoprotein)	Decrease in the incidence of infection, as manifested by Febrile Neutropenia, when receiving myelosuppressive anticancer drugs
Spevigo^TM^	Spesolimab-sbzo	Monoclonal Antibody	Pustular psoriasis flares
Tecvayli^TM^	Teclistamab-cqyv	Bispecific Monoclonal Antibody	Relapsed/refractory multiple myeloma
Tzield^TM^	Teplizumab-mzwv	Monoclonal Antibody	Delay the onset of stages 2/3 type 1 diabetes
Vabysmo^TM^	Faricimab-svoa	Bispecific Monoclonal Antibody	Neovascular age-related macular degeneration/diabetic macular edema
Xenpozyme^TM^	Olipudase alfa-rpcp	Enzyme	Non-central nervous system (CNS) manifestations of acid sphingomyelinase deficiency

^a^ Trade name used in the USA.

## Data Availability

Not applicable.

## References

[1] de la Torre B.G., Albericio F. (2022). The Pharmaceutical Industry in 2021. An Analysis of FDA Drug Approvals from the Perspective of Molecules. Molecules.

[2] U.S. Food and Drug Administration (FDA). https://www.fda.gov/drugs/development-approval-process-drugs/new-drugs-fda-cders-new-molecular-entities-and-new-therapeutic-biological-products.

[3] U.S. Food and Drug Administration (FDA). https://www.fda.gov/drugs/new-drugs-fda-cders-new-molecular-entities-and-new-therapeutic-biological-products/novel-drug-approvals-2022.

[4] U.S. Food and Drug Administration (FDA). https://www.fda.gov/vaccines-blood-biologics/development-approval-process-cber/2022-biological-license-application-approvals.

[5] U.S. Food and Drug Administration (FDA). https://www.fda.gov/emergency-preparedness-and-response/counterterrorism-and-emerging-threats/coronavirus-disease-2019-covid-19.

[6] https://investors.biogen.com/news-releases/news-release-details/eisai-presents-full-results-lecanemab-phase-3-confirmatory.

[7] https://aspr.hhs.gov/COVID-19/Treatments/Pages/Possible-Treatment-Options-for-COVID19.aspx.

[8] https://www.cdc.gov/coronavirus/2019-ncov/your-health/treatments-for-severe-illness.html.

